# Scaling Chromosomes for an Evolutionary Karyotype: A Chromosomal Tradeoff between Size and Number across Woody Species

**DOI:** 10.1371/journal.pone.0144669

**Published:** 2015-12-14

**Authors:** Guolu Liang, Hong Chen

**Affiliations:** 1 Department of Horticulture, College of Horticulture and Landscape Architecture, Southwest University, Chongqinq, China; 2 Department of Botany, College of Horticulture and Landscape Architecture, Southwest University, Chongqing, China; University of Science and Technology of China, CHINA

## Abstract

This study aims to examine the expected scaling relationships between chromosome size and number across woody species and to clarify the importance of the scaling for the maintenance of chromosome diversity by analyzing the scaling at the inter- & intra-chromosomal level. To achieve for the goals, chromosome trait data were extracted for 191 woody species (including 56 evergreen species and 135 deciduous species) from the available literature. Cross-species analyses revealed a tradeoff among chromosomes between chromosome size and number, demonstrating there is selective mechanism crossing chromosomes among woody species. And the explanations for the result were presented from intra- to inter-chromosome contexts that the scaling may be compromises among scale symmetry, mechanical requirements, and resource allocation across chromosomes. Therein, a 3/4 scaling pattern was observed between total chromosomes and m-chromosomes within nucleus which may imply total chromosomes may evolve from more to less. In addition, the primary evolutionary trend of karyotype and the role of m-chromosomes in the process of karyotype evolution were also discussed.

## Introduction

Chromosomes are the carriers of genetic material in the nucleus, and chromosome changes provide the basis of speciation [1–3]. In turn, the chromosomal complement, that is, the karyotype, is the highest level of structural and functional organization of chromosomes [4]; karyotypic properties are conferred by evolutionary changes [5] that result from natural selection [6] and may substantially influence plant performance [7]. For several decades, karyotypes have thus received intense attention as a prime cytological trait in empirical and theoretical research.

About the karyotypic evolution, Stebbins (1971) [8] has ever argued that asymmetric karyotypes are secondary and newer features compared with symmetric karyotypes. This hypothesis has achieved a certain degree of success due to its agreement with the "minimum interaction hypothesis", i.e., karyotype evolution generally tends to increase the number of acrocentric chromosomes to reduce occurrences of harmful mutations [9]. Possibly for this reason, the formation of metacentric chromosomes (m-chromosomes) is viewed as a "rare back-eddy" [10], something generated at random similarly to a backward source for karyotype evolution. If true, it would be deduced that there would be a scaling (i.e., a proportional size-dependent phenomenon, see [11, 12]) relationship between karyotype asymmetry and the number of m-chromosomes (m-CNs), since that the karyotype asymmetry index (KAI) is in its essence a parameter of size on chromosome arm length measured by the chromosome size or/and the centromere position [5]. This intra-cellular scaling of chromosome size (i.e., asymmetry index in this study) vs. number can also be based on the law of the constancy of the DNA content (see references in [13]) as observed by [14] and was indicated by the universal scaling laws of life (e.g., [15]).

Usually, karyotype symmetry can be changed by lengthening one arm at the expense of the other [8] such that the nucleus may invest more DNA in longer arms. Alternatively, symmetry may also be largely shaped by the numbers of different types of chromosomes due to the scaling: a nucleus with more m-chromosomes will appear more symmetric. In addition, scaling is perceived as size-dependent feedbacks in biology [12], or more specifically, a positive or negative proportional (scaling) regression relationship between biological variables [11]. And, the negative one is called tradeoff which plays a major role in diversity (see p.42 in [16]) due to the fact that advantages are offset by compensating disadvantages [17]. Determining the size-dependent relationship of chromosome size/ number inside a nuclear system is therefore helpful for understanding the evolution of chromosome size and of karyotype symmetry itself.

Just as meaningful as the phylogenic examination within a related plant group is, it is also interesting to compare the chromosomal properties across different taxa so to better understand the patterns of karyotype evolution and its underlying mechanisms, and significance for diversification and speciation [4]. To date, however, a positive or negative correlation between karyotype asymmetry and the acro-/meta-centric chromosome number have never been rigorously tested among plant chromosomes across a wide range of species. Hence, detailed knowledge of that is still lacking, although the importance of the mechanisms underlying intracellular chromosome scaling has been stressed recently (e.g., [12, 18]). Presumably for above reasons, the generality of the derived nature of the asymmetry has been questioned since it was postulated [5, 19, 20].

One reason for this situation may be that it is not clear how an addition or subtraction to a chromosome is apportioned between the two arms of the chromosome [21], which allocation is a mechanism that may underlie the scaling between karyotypic asymmetry and the number of acro-/meta-chromosomes. Assuming that each chromosome arm has the same increment in length because the chromosomes in the chromosome complement may vary together in a correlated fashion [6], then the symmetry of a chromosome would be altered because an equal addition to the two arms of an individual chromosome would cause a more symmetrical chromosome and vice versa for the equal subtraction to a more asymmetrical chromosome, as shown below:

when a>b, Δ*l* is a chromosome fragment of a length of *l*, then

$$\frac{a+\Delta l}{b+\Delta l}<\frac{a}{b}\;\frac{a+\Delta l}{b+2\Delta l}\ll \frac{a}{b}\;\frac{a-\Delta l}{b-\Delta l}>\frac{a}{b}$$ Eq I

Moreover, comparing the variations in chromosomal traits between two species groups sharing similar chromosomal architectures may be vital to understand the overall evolutionary direction of symmetry. For example, given Stebbins’ hypothesis, we may reasonably hypothesize that the karyotype of deciduous species (D-group) are more asymmetric than that of evergreen species (E-group) since the deciduous species are commonly accepted as newly evolved from the evergreens which results from divergent evolution as evidenced by the differences in resource utilization and geologic occurrence age on earth between the two broad-leaved species groups (e.g., [22, 23]). Meanwhile, it may also be predicted that the deciduous species would have fewer m-chromosomes according to the "minimum interaction hypothesis". Thus, according to the above two points, the scaling relationships of karyotype asymmetry vs. m-chromosome number among the two species groups might differ: the karyotype asymmetry of the deciduous species would be larger and with fewer m-chromosomes than in evergreen species.

In the current study, we first tested the predicted scaling relationships of chromosome size/number at the interchromosomal level across 191 wild species, and compared the two plant groups to identify selective mechanism underlying the karyotype evolution of dicot woody species; and from this comparison, we obtained evidence about the varying characteristics of the asymmetry. Second, the scaling connections between long- and short-arms at the intrachromosomal level were examined so to found the mechanisms underlying the evolutionary trends in karyotypic asymmetry and thus to clarify the scaling relationship between m-chromosomes and the evolution of karyotypes and as well.

## Materials and Methods

### Ethics statement

The dataset used in this study was collected from published books. The research did not involve measurements on humans or animals. We have no commercial interests or conflicts of interest in performing this work.

### Data sources and materials

The chromosome data of the 191 wild woody species employed in the current study were collected from two series of books [24]. These books contained many chromosomal traits for these species, including ploidy, number per nucleus (TCN), relative length of arms per individual chromosome (e.g., short-arm length (SL) and long arm length (LL)), and base number, which data can satisfy the requirements of chromosome traits of species for the quantitative and qualitative data analyses in this study. And all the species in these books were measured according to the standard of the [25] reported which stated that 30 cells were required for the measurement of chromosome number and >85% of the data must be consistently matched, and 5 cells required for karyotypic analyses. Karyotypic analysis method was referred to [26] and [25], and karyotypic classification followed to [8]. There were also comprehensive karyotypic features of a species examined, such as formula, nombre fundamental, KAI, constitution of relative length, and so on. The chromosomal traits of the 191 wild woody species used to perform the data analyses included the widest possible range of taxa and life forms (tree and shrub species: 135 deciduous species and 56 evergreen species; see the S1 Table).

The chromosome length used in this study was relative length which was calculated according to a formula [26] considering the ratio of the chromosome length to the total length. The arm ratio was the length of long arm relative to that of the short arm, and m-chromosome was defined as the chromosomes whose arm ratio ranged from 1.00 to 1.70 using the nomenclature for centromeric position on chromosomes [26]; this nomenclature includes M- and m-chromosome, which indicate that a centromere is located at the median- & middle-point, respectively. Considering the concept karyotype of asymmetry in the current study depended on the comparison at interchromosomal level (c.f., [27, 28]) rather than intrachromosomal one, we adopted KAI in our data analyses, consistent with [8], which KAI = AsK% = L/(L+S) = total relative length of long arms (L) in a chromosome set × 100 / total length of a chromosome set [29] or the identical AsI% ([30]).

### Data analysis

In this study, we adopted the method of allometric analysis introduced by Niklas ([11, 31]) for examining the scaling relationship of chromosomal traits at the inter- and intra-chromosome level. As is known, scaling relationship is given by the slope of the regression and the y-intercept of the regression curve governs its proportionality [11]. The bivariate scaling relationship of one variable against another was examined with standardized major axis tests & routines (SMA, [32]). If one variable scales proportionally with respect to another the scaling is isometric relationship; if it scales disproportionally, that is allometric one.

For improvement of the normality of variables, the data were log_10_-transformed prior to analysis. The bivariate line-fitting methods used included model II regression and a better method, 'reduced major axis' (RMA). The F-statistic was calculated to compare the sums of squares if a common slope was justified amongst several groups; then, a Wald statistic was used to compare several elevations that were fitted with MA or SMA lines that have a common slope [32]. If two groups of data with a common slope had no difference in elevation, their shift along the axis was subsequently examined using a Wald test for the location of a point along the fitted axis [33]. Phylogenetic independent contrast (PIC) analyses was not conducted in the current study because data from a wide range of species were used in this study; according to the view of [34], there might be little phylogenetic effect on the results and conclusions for this wide range of species.

## Results

### 1. The cross-species distribution of chromosome size and number

**Fig 1:**
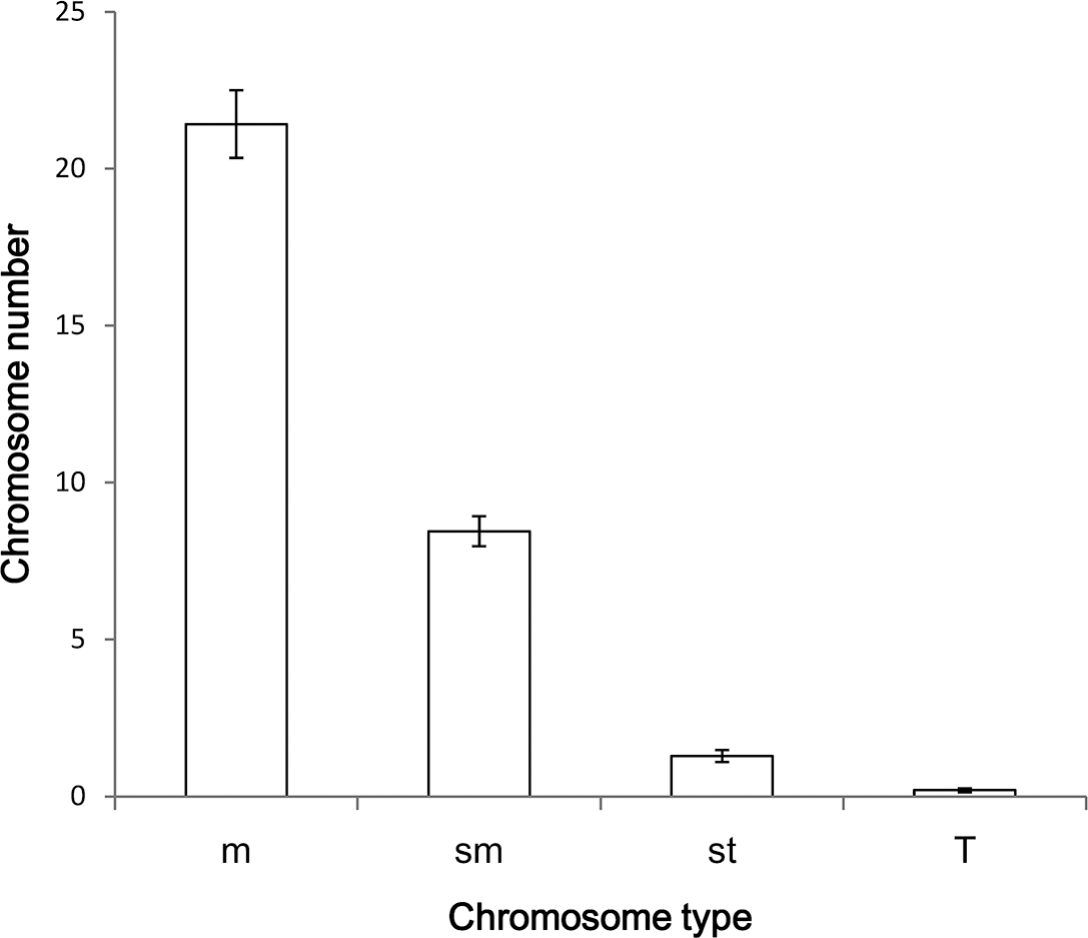
Frequency distribution of the number means of different chromosome types for the 191 woody broad-leaved species, which were averaged within the same type of chromosomes across species. Wherein (B), m, sm, st and T were m-, sm-, st- and T-chromosome, respectively. ± bars indicated standard error.

The karyotype asymmetry index, which actually can be viewed as chromosome size, varied among the 191species. The average karyotype asymmetry index was 61.815. Itranged from 53.38 of *Crataegus kansuensis* to 74.92 of *Ulmus pumila*, increasing ∼ 1.4 times. The means of chromosome number across species varied most among the four types of m-, sm-, st- and T-chromosome, and it ranged from 0.69% of T-chromosome to 68.3% of m-chromosome, covering close to ten times. The m-chromosomes accounted for nearly 70% of the chromosomes across species, while T-chromosome did for less than 1% (Fig 1). The above two results showed that the frequency distribution patterns of chromosomes was generally right-skewed.

### 2. The asymmetry index vs. m-chromosome number relationship

There was a significant relationship between the asymmetry index and the m-CN across all species (n = 191, r^2^ = 0.301, p < 0.001, slope = -0.097 [-0.109–0.086]) in the pooled data set. This result showed that KAI generally scaled negatively with m-CN.

**Fig 2:**
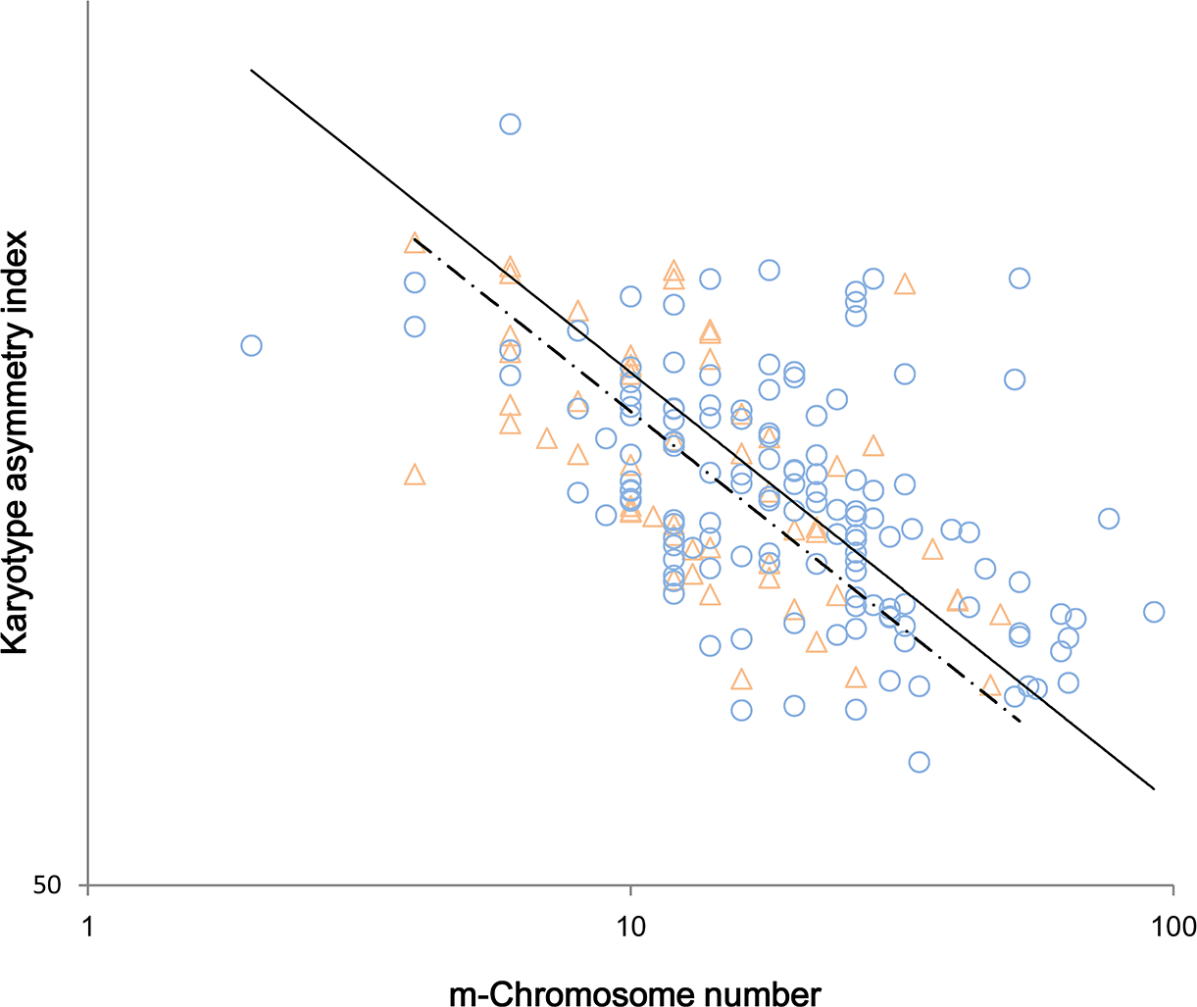
Cross-species scaling relationship of karyotype asymmetry vs. m-chromosome number among deciduous species (D-group) and evergreen species (E-group). There are two regression lines of the reduced major axis (RMA) in the graph for the D-group (triangles and the solid line) and for the E-group (circles and the dashed line), respectively.

When 191 species were grouped as evergreen and deciduous species, tests for slope heterogeneity indicated that the scaling relationship did not significantly differ between the two species groups (p = 0.517), and a common slope = -0.099 was found. Next, an obvious shift in elevation (y-intercept) between the groups was observed (r^2^ = 0.273, p <0.001, slope = -0.097 [-0.112, -0.084] for the D-group; r^2^ = 0.346, p<0.001, slope = -0.107 [-0.132, -0.085] for the E-group) with a significant elevational decline from the former (y-intercept_*D*_ = 1.917) to the latter (y-intercept_*E*_ = 1.908; Fig 2). Moreover, there was also a significant shift along the common slope (stat = 8.595, p = 0.003; XgrandY of the D-group = 1.266 > 1.181 of the E-group value, where Y and X were KAI and X m-CN, respectively).

Thus, there is a tradeoff generally between KAI and m-CN, indicating that KAI would decrease across all species when m-CN increases. Moreover, at a given m-CN value, the deciduous species are more asymmetric than the evergreens, and the deciduous species also have more m-chromosomes than the evergreens. The deciduous species thus tend to be more asymmetric than the evergreen species in addition to possessing more m-chromosomes.

### 3. The scaling between the lengths of short vs. long arm across chromosomes

**Fig 3:**
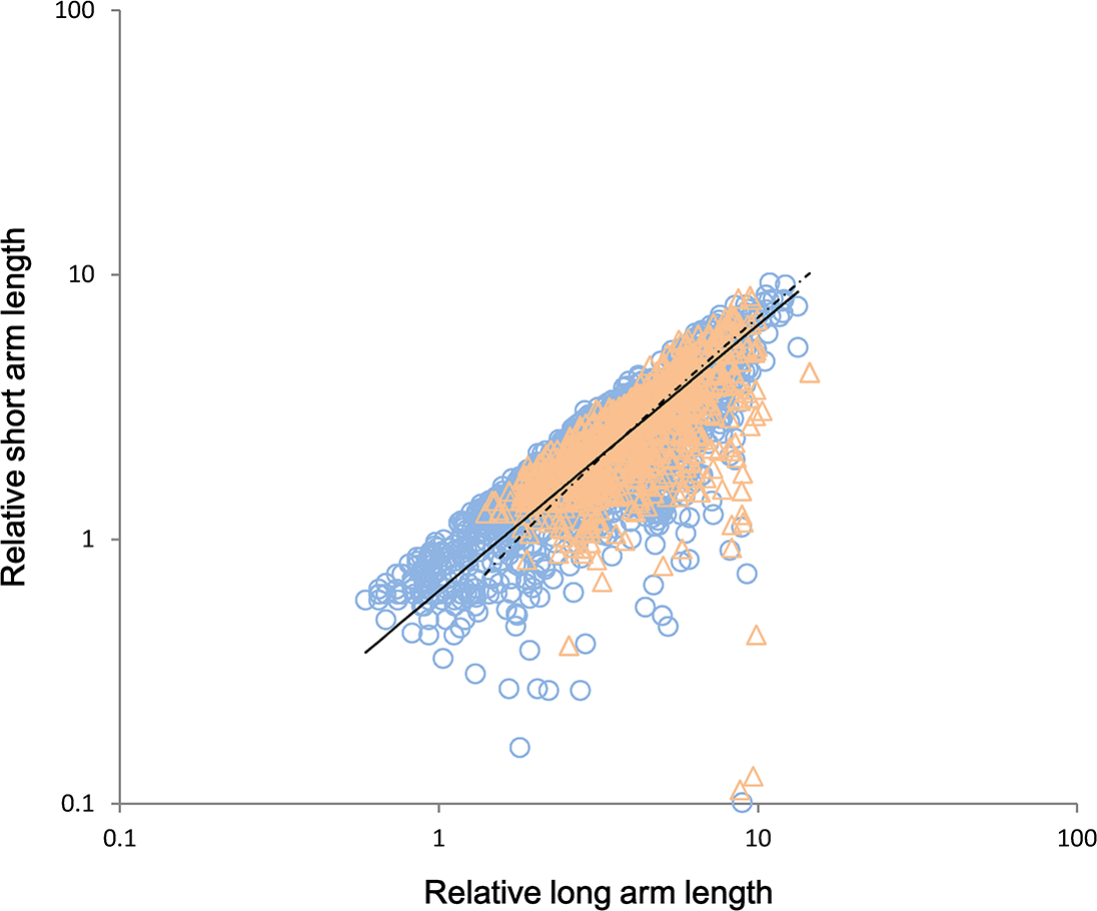
Cross-species scaling relationship of relative short arm length vs. relative long arm length among chromosomes of both deciduous species (D-group) and evergreen species (E-group). There are two regression lines of the reduced major axis (RMA) in the graph for the D-group (triangles and the solid line) and for the E-group (circles and the dashed line), respectively.

Although the SLs proportionally scaled with the LLs across all chromosomes (n = 3051, r^2^ = 0.599, p<0.001) in the pooled data with a slope = 1.018 close to 1.0 [0.995, 1.041] (p = 0.122), the scaling correlation of evergreens markedly differed from that of the deciduous species (post-hoc test: p = 0.001). The SLs of the D-group scaled proportionally as LLs increased with slope = 1.006 [0.982, 1.031] (n = 2238, r^2^ = 0.660), whereas the SLs of the E-group did not scale proportionally with the LLs (n = 801, r^2^ = 0.364, p<0.001) showing an allometric exponent of 1.119 [1.059, 1.182] which is significantly larger than 1.0 (p<0.001). That was, as the LLs increased, the SLs of the E-group plants increased faster than those of D-group ones (Fig 3).

The above results showed that the incremental changes in length markedly differed among the two groups. The SLs of the D-group species maintained a proportionally increasing pace with the increasing LLs at the intrachromosomal level, but that of E-group followed an allometrically faster pace. Consequently, with the increasing of long arm the E-group species tend to increase their symmetry faster than the D-group ones according to Eq I.

### 4. The relationship between m-CN and TCN

**Fig 4:**
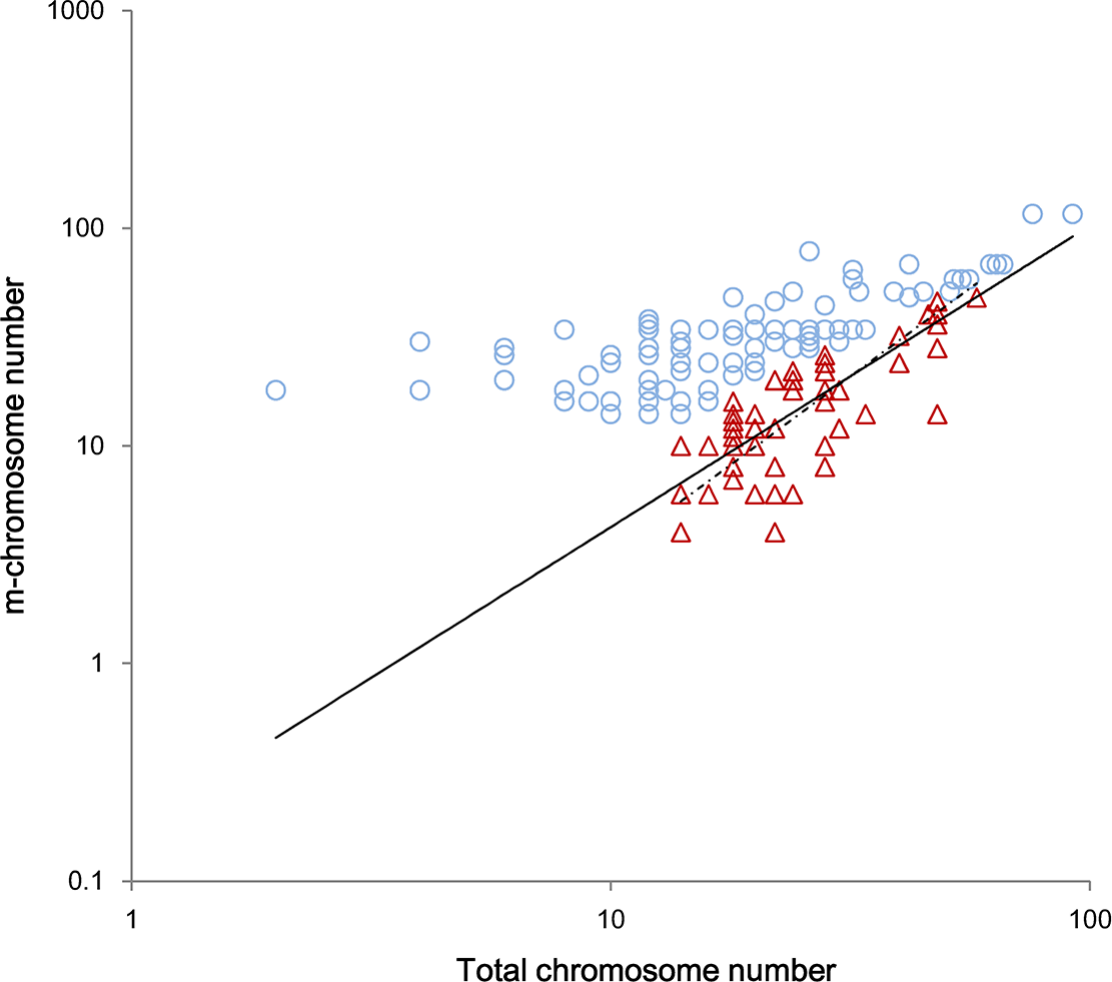
Cross-species scaling relationship of m-chromosome number vs. total chromosome number among deciduous species (D-group) and evergreen species (E-group). There are two regression lines of the reduced major axis (RMA) in the graph for the D-group (triangles and the solid line) and for the E-group (circles and the dashed line), respectively.

The m-CN was allometrically related to TCN in the pooled data set (r^2^ = 0.619, p<0.001) with a common slope (= 1.449 [1.326, 1.583]) that did not depart from the value of 1.5 (p = 0.402) for the two functional groups (Fig 4); the allometry exponent = 0.692 [0.632, 0.760] did not depart from 3/4 (p = 0.094) when the bivariate connection was expressed as TCN vs. m-CN. There was no notable shift in the y-intercept (p = 0.134). However, a shift along the common slope was significant: the XgrandY of the D-group = 1.446 < E-group = 1.461, where Y and X were m-CN and TCN, respectively, or the XgrandY of the D-group = 1.248 > E-group = 1.226, where Y and X were TCN and m-CN, respectively.

These results showed that the scaling relationship of m-CN vs. TCN was allometric with a scaling exponent of 1.5 or 3/4, indicating m-CN may increase at a 1.5 times faster pace than the increase in TCN or that the TCN increased at a 3/4 slower pace than the m-CN did. Additionally, the E-group species may have more chromosomes in their nucleus, but the D-group has more m-chromosomes.

## Discussion

Consistent with our prediction, karyotype asymmetry correlated negatively with m-chromosome number across species, indicating a tradeoff between size (i.e., karyotype, also see the following below) and number. In addition, the evolutionary tendency of karyotype asymmetry fits the hypothesis of Stebbins in 1971 [8], as evidenced by our result of a more asymmetric karyotype in deciduous species. Nevertheless, the more asymmetric the deciduous species was the more m-chromosomes there were (Fig 2), demonstrating that the increased asymmetry was offset to some degree by the more m-chromosomes. This size/number tradeoff may be achieved through a size-dependent allometry of intrachromosomal length and a density-dependent scaling from intra- to inter-chromosomes.

### 1. The evolutionary direction of karyotype symmetry

There was no obvious heterogeneity in the scaling relationship of asymmetry vs. m-chromosomes between the two groups shown by their common slope of the scaling; however, there were obvious differences both in the elevation and in the x-axis along their common slope. This finding indicates that the woody species do not exhibit much differentiation between the two groups on the whole, implying a limited asymmetry in the evolution of karyotypes. A more detailed discussion of this idea is highlighted in the following sections.

Furthermore, at a given number of m-chromosomes, the deciduous species were more asymmetric than the evergreens, as demonstrated by the higher y-intercept of the D-group (Fig 2). Thus, chromosomes do indeed evolve in a direction from symmetry to asymmetry as a new evolutionary feature, at least among woody species, considering the common sense of the deciduous species are more evolutionary than the evergreens. This assertion is, consistent with the observations that many genera show increasing asymmetry and thus it may be a widespread karyotypic feature in Angiosperms [8, 35].

### 2. The mechanism underlying the evolution of karyotype symmetry

At the intrachromosomal level, the scaling correlation of arm size in evergreens was markedly different from that in deciduous species (Fig 3), indicating the differing increment of chromosome segments. As the long-arm length increased, the short arms of the D-group scaled proportionally, whereas that of the E-group plants increased in an allometric, rather than isometric, manner. As a result, the karyotype was more asymmetric as the long-arm length increased in the D-group species but was more symmetric in the E-group, as indicated by the analyses of the arm size in the introduction section (Eq I). Furthermore, this difference may be the mechanism responsible for the previously observed cases of symmetry in deciduous species and vice versa for the evergreens (Fig 2). This explanation is also partially in accordance with the notion, especially for the deciduous species, that the amount of nuclear DNA within genera is typically changed by equal increments to each chromosome [36].

Note that, symmetry requires more chromosome fragments with the increase of long arm length but asymmetry does less, logically emerged from Eq I; thus, more microtubulins are required to satisfy the demands for the materials to ensure accurate genome distribution during anaphase during the process of cell formation. However, the amounts of both chromosome fragments and microtubulins are not limitless whether in evergreens or in the deciduous species because of constancy of the amount of DNA and because of the idea of meiotic spindle limitation [18]. And hence it is not surprising for species to evolve a mechanism sizing chromosomes towards an asymmetric karyotype as observed in the deciduous species.

### 3. The role of m-chromosomes in karyotype evolution

In this context, the significance of deciduous species having more m-chromosomes along the common slope is equally important (Fig 2); this finding is inconsistent with our prediction because it indicates that deciduous species may contain more m-chromosomes, although these species were more asymmetric. This result implies that the quantity of m-chromosomes in a nucleus is a negative agent at the interchromosomal level that reduces the over-development of asymmetry [27, 28] and thus provides a "symmetry-preserving" trend of karyotypic variations across species. Although there was a typically size-dependent evolution at the intrachromosome level toward asymmetry (Fig 2), the tendency may be density-limited by the increased amount of m-chromosomes at the interchromosome level. We suggest the increasing m-CNs are more like a negative feedback mechanism, i.e., a selective pressure favoring asymmetry over symmetry to prevent the production of an over-asymmetric karyotype, that is, m-chromosomes exert an antiasymmetric effect on karyotype; this proposal complies well with the "scale symmetry" theory in biology [37, 38].

In this sense, m-chromosomes have a special significance for a balanced karyotype; in contrast to the view of “rare back-eddies”, m-chromosomes provide an effect that allows chromosome diversity or coexistence just as shown by the remarkably right-skewed distribution of chromosome number (Fig 1; [39]), and hence species diversity, to be sustainable according to the arguments of [17]. Because the karyotype is the highest structural organization, the karyotype is very likely affected by both the size and number from the two levels. Negative correlations between chromosome size and number are still rarely reported and are reported even less for tree species, although Wise et al. (2009)[40] have published a study with a little similar results in terms of the scaling of chromosomes (telomere length vs. chromosome arm) in human cells. Nevertheless, such a positive scaling among chromosome-element sizes is not a tradeoff after all. In this regard, we show a tradeoff for the first time and provide evidence for the existence of intra-nucleus scaling (c.f., [12]).

Two possible features may account for this phenomenon. First, this feature reflects the fact that the nucleus may have evolved a mechanism that constrains chromosome structure [10, 41] to minimize energy requirements and thus to build an almost-optimized karyotype according to the minimization principle [15]. The optimal ranges for the upper and lower tolerance limits for chromosome size have been suggested [10 p111], [41], and reaching these values indicates profit maximization and optimal resource allocation [42] when resources (e.g., chromosome segments) are limited in a nucleus. This feature is particularly important when assuming that karyotype asymmetry is an evolutionary benefit and that the increased number of m-chromosomes is a cost (c.f., [39]) that is necessarily paid by deciduous species for their karyotype evolution according to the tradeoff theory.

Second, another reason for the necessity of more m-chromosomes in a nucleus may be the requirements of spindle fiber biomechanics. For a spindle fiber, dragging a chromatid with two arms of equal length in fluid may require less energy than dragging non-balanced ones; in the latter case, extra torque against chromosome sliding must be placed on a centromere.

### 4. The relationship between m-chromosomes and total chromosomes

The m-CNs in a nucleus was positively related to the total chromosome number with an allometric exponent of 1.5 (Fig 4), or, the TCNs was correlated with the m-CNs with a 3/4 scaling; additionally, the deciduous species had more m-chromosomes, whereas the evergreens had more total chromosomes. The above may provide a clue about the evolution of the total chromosomes number. For instance, we can reasonably infer that the evolutionary direction of total chromosome number should be from more to less, both because evergreens had more chromosomes in their nuclei and because increases in TCN will trigger an increase in m-chromosomes at a 1.5-fold faster rate. Thus, it is fairly costly for a species to increase the chromosomal number in its nucleus. As another example, a marginal increase in m-CN may in turn lead to only 3/4 increase in TCN, consistent with the 3/4 rule [43], meaning that species with one more m-Ch has only 3/4 more TCNs. This “diminishing returns” in the chromosome number may imply the TCN may be an “inert” component among chromosomes that results from a tradeoff between ancestral morphological traits (c.f., [43]). Again, this relationship is also in agreement with the 3/4 exponent in [44], who stresses that the scale symmetry directly accounts for power laws [45] when the quantitative relation between the part (e.g., m-CN) and the whole (e.g., TCN) is considered.

## Conclusion

In summary, the tradeoff between m-chromosome number and karyotype asymmetry indicates the existence of a scaling relationship between the size and number of chromosomes within a nucleus across woody species. The tradeoff may be significant as an evolved mechanism that prevents the over-asymmetry of a karyotype for the sake of the maintenance of chromosome diversity, and it may also be a biomechanical limit of spindle fibers. The result holds potential both to indicate the evolutionary direction of size-dependent karyotype symmetry and to imply a density-dependent symmetry as well. Perhaps, a stable, equilibrated karyotype is a more appropriate choice for a nucleus and an even more appropriate choice for a species. The result may also prove the generality of scaling theory [46] at the chromosome level, a persistent theme in biology; thus, the findings may contribute to comparative studies of karyotypic morphogenesis among species and to the understanding of the chromosome evolutionary path towards an optimal karyotype.

## Supporting Information

S1 Table The chromosome traits for the 191 species used in the current study. LF = life form, D = deciduous species, E = evergreen species, TCN = total chromosome number, m-CN = m-chromosome, KAI = karyotype asymmetry index, LcN = long chromosome number, ScN = short chromosome number.
(PDF)
