# Supplementary material for: Scaling Chromosomes for an Evolutionary Karyotype: A Chromosomal Tradeoff between Size and Number across Woody Species
Source: PLoS One. 2015 Dec 14;10(12):e0144669. doi: 10.1371/journal.pone.0144669 (PMC4684206; doi:10.1371/journal.pone.0144669)
Supplement: S1 Table — LF = life form, D = deciduous species, E = evergreen species, TCN = total chromosome number, m-CN = m-chromosome, KAI = karyotype asymmetry index, LcN = long chromosome number, ScN = short chromosome number. (PDF) [file pone.0144669.s001.pdf]

S1 Table

Species	Family	LF	TCN	m-CN	KAI	LcN	ScN
Acacia catechu	*Mimosaceae*	D	26	10	65.32	10	16
Acanthopanax senticosus	*Araliaceae*	D	48	18	65.06	26	22
Acanthopanax trifoliatus	*Araliaceae*	E	48	14	67.17	26	22
Actinidia arguta	*Actinidiaceae*	D	116	92	57.82	52	64
Actinidia callosa	*Actinidiaceae*	D	58	52	69.02	20	38
Actinidia melliana	*Actinidiacea*	E	58	48	57.74	32	26
Actinidia chinensis	*Actinidiaceae*	D	58	52	57.17	24	34
Actinidia eriantha	*Actinidiaceae*	D	58	52	57.06	26	32
Actinidia hemsleyana	*Actinidiaceae*	D	58	54	55.58	26	32
Actinidia kolomikta	*Actinidiaceae*	D	58	52	58.74	28	30
Actinidia latifolia	*Actinidiaceae*	D	58	56	55.49	28	30
Actinidia macrosperma	*Actinidiaceae*	D	116	76	60.75	54	62
Actinidia polygama	*Actinidiaceae*	D	58	32	61.87	28	30
Ailanthus altissima	*Simaroubaceae*	D	64	32	65.6	30	32
Albizzia julibrissin	*Leguminosae*	D	26	12	64.4	14	12
Ambroma augusta	*Sterculiaceae*	E	20	10	65.6	10	10
Amygdalus persica	*Rosaceae*	D	16	14	69	4	12
Amygdalus mira	*Rosaceae*	D	16	10	64.17	6	10
Amygdalus mongolica	*Rosaceae*	D	16	9	60.86	5	11
Amygdalus tangutica	*Rosaceae*	D	16	10	61.97	6	10
Aralia chinensis	*Araliaceae*	E	24	20	60.4	10	14
Ardisia caudata	*Myrsinaceae*	E	46	40	58.21	24	22
Armeniaca sibirica	*Rosaceae*	D	16	10	61.65	4	12
Bauhinia blakeana	*Leguminosae*	E	28	16	62.89	12	16
Bauhinia pernervosa	*Leguminosae*	E	28	22	60.32	16	12
Bixa orellana	*Bixaceae*	E	14	10	61.01	6	8
Broussonetia papyrifera	*Moraceae*	D	28	14	64.53	10	18
Chaenomeles speciosa	*Rosaceae*	D	34	20	65.68	18	16
Cajanus cajan	*Fabaceae*	E	22	20	57.9	14	8
Callicarpa bodinieri	*Verbenaceae*	D	40	20	65.49	18	22
Canarium pimela	*Burseraceae*	E	48	46	55.62	18	30
Canarium tonkinense	*Burseraceae*	E	48	36	59.79	22	26
Carya cathayensis	*Juglandaceae*	D	32	26	59.67	14	18
Cassia siamea	*Caesalpiniaceae*	E	28	10	65.86	12	16
Cassia sophera	*Leguminosae*	E	14	4	62.21	6	8
Castanea crenata	*Fagaceae*	D	24	16	61.9	10	14
Castanea dentata	*Fagaceae*	D	24	18	61.46	10	14
Castanea mollissima	*Fagaceae*	D	24	18	69.33	6	18
Castanea mollissima	*Fagaceae*	D	24	14	60.12	10	14
Castanea seguinii	*Fagaceae*	D	24	14	62.25	10	14
Cerasus tomentosa	*Rosaceae*	D	16	12	58.38	8	8

Cerasus humilis	*Rosaceae*	D	16	8	61.6	4	12
Cerasus serrulata	*Rosaceae*	D	16	12	60.13	6	10
Cinnamomum bodinieri	*Lauraceae*	E	24	18	59.33	10	14
Cipadessa baccifera	*Meliaceae*	E	28	22	60.48	12	16
Citrus reticulata	*Rutaceae*	E	18	13	59	9	9
Citrus chuana	*Rutaceae*	E	18	12	69.01	6	12
Citrus daoxianensis	*Rutaceae*	E	18	10	62.51	10	8
Citrus hongheensis	*Rutaceae*	E	18	7	63.41	6	12
Citrus sulcata	*Rutaceae*	E	18	14	59.83	9	9
Citrus tangerita	*Rutaceae*	E	18	10	61.16	6	12
Clausena lansium	*Rutaceae*	E	18	16	55.8	6	12
Clematis aethusifolia	*Ranunculaceae*	D	16	10	68.36	8	8
Clematis chinensis	*Ranunculaceae*	E	16	10	66.26	8	8
Clematis finetiana	*Ranunculaceae*	E	16	6	69.21	8	8
Clematis florida	*Ranunculaceae*	E	16	10	65.93	10	6
Clematis heracleifolia	*Ranunculaceae*	D	16	10	65.84	10	6
Corylus heterophylla	*Corylaceae*	D	22	20	55	10	12
Crataegus altaica	*Rosaceae*	D	68	62	56.62	36	32
Crataegus cuneata	*Rosaceae*	D	34	26	60.22	14	20
Crataegus hupehensis	*Rosaceae*	D	34	28	60.76	22	12
Crataegus kansuensis	*Rosaceae*	D	34	34	53.38	18	16
Crataegus maximowiczii	*Rosaceae*	D	51	51	65.41	27	24
Crataegus pinnatifida	*Rosaceae*	D	34	28	58.03	14	20
Crataegus pinnatifida	*Rosaceae*	D	51	51	55.27	30	21
Crataegus sanguinea	*Rosaceae*	D	68	64	57.02	30	38
Crataegus scabrifolia	*Rosaceae*	D	34	26	59.4	14	20
Crataegus songorica	*Rosaceae*	D	68	66	57.61	28	40
Crataegus wilsonii	*Rosaceae*	D	34	32	56.92	14	20
Croton tiglium	*Euphorbiaceae*	E	20	6	63.91	10	10
Datura stramonium	*Solanaceae*	E	24	18	58.88	12	12
Dichroa febrifuga	*Saxifragaceae*	D	20	6	65.56	10	10
Diospyros discolor	*Ebenaceae*	E	30	18	61.63	14	16
Diospyros lotus	*Ebenaceae*	D	32	18	61.34	14	18
Diospyros rhombifolia	*Ebenaceae*	D	30	26	68.16	14	16
Diospyros glaucifolia	*Ebenaceae*	D	30	30	57.68	10	20
Diospyros oleifera	*Ebenaceae*	D	30	22	59.31	12	18
Dipteronia dyerana	*Aceraceae*	D	22	14	60.62	8	14
Dracaena cambodiana	*Liliaceae*	E	40	24	62.48	16	24
Duranta repens	*Verbenaceae*	E	34	14	67.05	18	16
Elaeagnus pungens	*Elaeagnaceae*	E	28	8	67.86	6	22
Flueggea suffruticosa	*Euphorbiaceae*	D	26	10	64.47	12	14
Forsythia suspensa	*Oleaceae*	D	28	12	64.02	12	16
Fortunella obovata	*Rutaceae*	E	18	11	60.83	11	7
Fortunella margaria	*Rutaceae*	E	18	13	59.76	8	10

Fraxinus chinensis	*Oleaceae*	D	46	22	64.16	22	24
Helicteres angustifolia	*Sterculiaceae*	D	18	16	59.55	8	10
Hippophae rhamnoides	*Elaeagnaceae*	D	24	20	57.47	8	16
Hodgsonia macrocarpa	*Cucurbitaceae*	E	18	10	60.97	8	10
Indigofera suffruticosa	*Leguminosae*	D	16	14	56.78	6	10
Indigofera zollingeriana	*Leguminosae*	D	20	12	63.29	10	10
Juglans cathayensis	*Juglandaceae*	D	32	26	62.02	12	20
Juglans mandshurica	*Juglandaceae*	D	32	26	61	18	14
Kadsura japonica	*Schisandraceae*	E	28	26	55.85	14	14
Leea macrophylla	*Leeaceae*	E	48	28	63.17	24	24
Liquidambar formosana	*Hamamelidaceae*	D	26	6	66.44	10	16
Lonicera japonica	*Caprifoliaceae*	D	18	2	66.6	8	10
Lycium chinense	*Solanaceae*	D	24	18	59.64	8	16
Macrocarpium officinale	*Cornaceae*	D	18	8	64.41	6	12
Mahonia fortunei	*Berberidaceae*	E	28	24	58.33	14	14
Malus formosana	*Rosaceae*	D	34	26	68.54	16	18
Malus asiatica	*Rosaceae*	D	34	30	57.91	16	18
Malus asiatica	*Rosaceae*	D	68	42	60.31	26	42
Malus asiatica	*Rosaceae*	D	34	22	62.21	14	20
Malus asiatica	*Rosaceae*	D	34	28	69.01	14	20
Malus halliana	*Rosaceae*	D	51	33	60.42	18	33
Malus kansuensis	*Rosaceae*	D	34	18	63.57	16	18
Malus komarovii	*Rosaceae*	D	34	32	57.39	20	14
Malus melliana	*Rosaceae*	D	34	30	55.74	14	20
Malus prunifolia	*Rosaceae*	D	34	30	60.17	16	18
Malus pumila	*Rosaceae*	D	34	26	58.27	18	16
Malus pumila	*Rosaceae*	D	34	26	60.07	16	18
Malus sieboldii	*Rosaceae*	D	51	39	60.4	21	30
Malus sieversii	*Rosaceae*	D	34	24	61.04	12	22
Malus sikkimensis	*Rosaceae*	D	68	62	57.75	34	34
Malus toringoides	*Rosaceae*	D	34	30	57.65	16	18
Malus transitoria	*Rosaceae*	D	34	12	63.27	18	16
Malus xiaojinensis	*Rosaceae*	D	68	64	55.68	26	42
Malus honanensis	*Rosaceae*	D	34	18	65.94	12	22
Malus hupehensis	*Rosaceae*	D	51	45	59.16	24	27
Malus ombrophila	*Rosaceae*	D	34	20	62.31	12	22
Malus prattii	*Rosaceae*	D	34	32	58.06	14	20
Malus yunnanensis	*Rosaceae*	D	34	20	62.34	18	16
Mangifera sylvatica	*Anacardiaceae*	E	40	32	68.84	16	24
Melia azedarach	*Meliaceae*	D	28	20	61.01	16	12
Morus cathayana	*Moraceae*	D	28	26	54.89	10	18
Morus mongolica	*Moraceae*	D	28	26	57.3	12	16
Mucuna sempervirens	*Fabaceae*	E	22	8	64.67	8	14
Murraya exotica	*Rutaceae*	E	18	12	58.83	10	8

Neolitsea menglaensis	*Lauraceae*	E	24	6	69.47	12	12
Oroxylum indicum	*Bignoniaceae*	D	28	26	58	14	14
Phellodendron amurense	*Rutaceae*	D	78	26	67.66	32	46
Plumbago zeylanica	*Plumbaginaceae*	E	28	18	63.43	12	16
Poncirus trifoliata	*Rutaceae*	D	18	13	59.82	12	6
Poncirus trifoliata	*Rutaceae*	D	18	12	59.9	8	10
Priotropis cytisoides	*Leguminosae*	D	16	12	58.94	8	8
Prunus mume	*Rosaceae*	D	16	12	60.59	6	10
Prunus simonii	*Rosaceae*	D	16	10	61.4	10	6
Prunus ussuriensis	*Rosaceae*	D	16	10	61.69	8	8
Prunus cerasifera	*Rosaceae*	D	16	12	59.45	8	8
Psidium guajava	*Myrtaceae*	E	22	6	64.54	8	14
Pyracantha fortuneana	*Rosaceae*	E	34	14	66.13	14	20
Pyrus betulaefolia	*Rosaceae*	D	34	22	61.62	14	20
Pyrus bretschneideri	*Rosaceae*	D	34	26	59.08	12	22
Pyrus bretschneideri	*Rosaceae*	D	34	8	67.13	14	20
Pyrus calleryana	*Rosaceae*	D	34	26	60.83	12	22
Pyrus pyrifolia	*Rosaceae*	D	34	20	61.88	16	18
Pyrus armeniacaefolia	*Rosaceae*	D	51	24	64.73	24	27
Pyrus hopeiensis	*Rosaceae*	D	34	16	64.35	14	20
Pyrus pashia	*Rosaceae*	D	34	18	63.46	14	20
Pyrus phaeocarpa	*Rosaceae*	D	34	22	61.29	12	22
Pyrus serrulata	*Rosaceae*	D	34	14	64.09	16	18
Pyrus sinkiangensis	*Rosaceae*	D	34	18	62.71	14	20
Pyrus xerophila	*Rosaceae*	D	34	22	62.83	16	18
Rauvolfia vericillata	*Apocynaceae*	E	22	12	63.34	10	12
Rauvolfia verticillata	*Apocynaceae*	E	22	4	70.36	10	12
Rhus chinensis	*Anacardiaceae*	D	30	14	65.57	14	16
Ribes rubrum	*Saxifragaceae*	D	16	16	56.99	6	10
Ribes burejense	*Saxifragaceae*	D	16	16	54.87	6	10
Ribes mandshuricum	*Saxifragaceae*	D	16	12	58.77	8	8
Ricinus communis	*Euphorbiaceae*	E	20	14	58.35	8	12
Rosa laevigata	*Rosaceae*	E	14	6	66.96	6	8
Rubus crataegifolius	*Rosaceae*	D	21	9	63.4	12	9
Rubus idaeus	*Rosaceae*	D	14	14	59.17	8	6
Rubus komarovi	*Rosaceae*	D	14	12	60.71	6	8
Rubus sachalinensis	*Rosaceae*	D	14	10	61.33	6	8
Rubus xanthocarpus	*Rosaceae*	D	14	10	62.86	6	8
Rubus idaeus	*Rosaceae*	D	21	18	59.34	9	12
Rubus parvifolius	*Rosaceae*	D	28	24	60.26	18	10
Salix myrtillacea	*Salicaceae*	E	28	16	64.23	10	18
Sambucus williamsii	*Caprifoliaceae*	D	36	12	68.06	20	16
Sarcandra glabra	*Chloranthaceae*	E	30	12	69.33	12	18
Schisandra chinensis	*Schisandraceae*	D	28	24	57.11	16	12

Solanum verbascifolinm	*Solanaceae*	D	24	10	64.84	10	14
Sophora davidii	*Leguminosae*）	D	18	12	63.14	8	10
Sophora flavescens	*Fabaceae*	D	18	12	64.42	10	8
Sophora tonkinensis	*Leguminosae*	E	18	8	62.87	8	10
Spiraea salicifolia	*Rosaceae*	D	18	4	67.27	8	10
Syzygium jambos	*Myrtaceae*	D	44	28	61.67	18	26
Terminalia chebula	*Combretaceae*	D	48	42	57.96	14	34
Thespesia lampas	*Malvaceae*	E	20	6	66.37	10	10
Toxicodendron vernicifluum	*Anacardiaceae*	D	30	4	68.88	14	16
Trachelospermum jasminoides	*Apocynaceae*	E	20	12	60.58	10	10
Ulmus pumila	*Ulmaceae*	D	28	6	74.92	14	14
Urena procumbens	*Malvaceae*	D	38	12	66.01	18	20
Vaccinium corymbasum	*Ericaceae*	E	48	40	58.16	24	24
Vaccinium vitis-idaea	*Ericaceae*	E	24	22	56.91	10	14
Viburnum utile	*Caprifoliaceae*	E	18	12	60.22	6	12
Vitis negundo	*Verbenaceae*	D	34	34	55.58	16	18
Vitis rotundifolia	*Verbenaceae*	D	34	16	64.06	16	18
Ziziphus jujuba	*Rhamnaceae*	D	24	16	62.2	10	14

3
